# Machine learning-based prediction of 30-day unplanned readmission risk in day surgery lung cancer patients after lobectomy or sublobectomy: a real-world study

**DOI:** 10.3389/fmed.2026.1771501

**Published:** 2026-02-20

**Authors:** Nafei Han, Chuanbo An, Huadi Yuan, Meijuan Lan, Xiaoyan Wu, Li Liu, Xiaowei Yu, Xiajuan Jiang, Liyan Gao, Jing Fang

**Affiliations:** 1 Department of Nursing, The Second Affiliated Hospital, Zhejiang University School of Medicine, Hangzhou, China; 2 Department of Big Data Health Science, School of Public Health, Zhejiang University School of Medicine, Hangzhou, Zhejiang, China; 3 Center for Rehabilitation Medicine, Rehabilitation & Sports Medicine Research Institute of Zhejiang Province, Department of Rehabilitation Medicine, Zhejiang Provincial People’s Hospital (Affiliated People’s Hospital), Hangzhou Medical College, Hangzhou, Zhejiang, China

**Keywords:** 30-day unplanned readmission, day surgery lung cancer, machine learning, random forest, unplanned readmission risk

## Abstract

**Background:**

Unplanned readmission within 30 days after lobectomy or sublobectomy for early stage lung cancer adversely affects patient recovery and healthcare costs. While machine-learning (ML) approaches offer potential for improved prediction, few models have been developed for day-surgery settings. This study aimed to develop and validate an ML-based model to predict 30-day unplanned readmission in lung cancer patients undergoing ambulatory lung resection.

**Methods:**

We included patients who underwent lobectomy or sublobectomy in a day-surgery pathway between December 2022 and January 2025. The least absolute shrinkage and selection operator (LASSO) was used for feature selection. Data were split into training (70%) and validation (30%) sets. Nine ML algorithms were trained and evaluated using area under the receiver-operating-characteristic curve (ROC-AUC), precision-recall AUC (PR-AUC), accuracy, decision-curve analysis (DCA), and calibration curves. Model interpretability was assessed with SHapley Additive exPlanations (SHAP).

**Results:**

After propensity-score matching, 380 patients were analyzed, including 111 with unplanned readmission. LASSO identified 12 predictive features: age, payment category, prothrombin time (PT), white-blood-cell count (WBC), hemoglobin, intraoperative blood loss, surgical approach, pathological diagnosis, tumor count, tumor size, occupational category, and forced expiratory volume in 1 s (FEV_1_). The random forest (RF) model performed best in the validation set (ROC-AUC = 0.939, accuracy = 0.825), showed favorable net benefit across threshold probabilities of 10–80%, and was well-calibrated. SHAP analysis indicated WBC, PT, hemoglobin, intraoperative blood loss, and “unknown” occupational category as the top five predictors; WBC, PT, and blood loss were positively associated with readmission risk.

**Conclusion:**

An RF-based model effectively predicted 30-day unplanned readmission after lung-cancer day surgery. The identified risk factors provide a basis for early stratification and targeted intervention, supporting optimized perioperative care in ambulatory settings.

## 1 Background

Lung cancer remains the leading cause of cancer-related mortality worldwide, with surgical resection as the primary curative approach for early stage disease (1). Lobectomy and sublobectomy (segmentectomy or wedge resection) are commonly performed, yet their comparative outcomes continue to be debated (2, 3). Although minimally invasive techniques and enhanced recovery protocols have reduced postoperative stay (4, 5), unplanned readmission remains a significant concern, associated with increased costs and poorer long-term prognosis (6).

In conventional inpatient settings, factors such as impaired lung function, open surgery, and prolonged hospitalization have been linked to higher readmission risk (7, 8). However, the rapidly expanding day surgery model, characterized by discharge within 1–2 days, poses distinct challenges. Early discharge may limit opportunities to detect evolving complications, potentially elevating readmission risk (9). Existing prediction tools, largely derived from traditional hospitalization cohorts, may not generalize to day surgery populations.

Machine learning (ML) offers advantages over conventional statistical methods in handling high-dimensional data, capturing non-linear relationships, and automating feature selection (10, 11). ML models have demonstrated superior performance in predicting postoperative complications (12), but their application to readmission risk after lung cancer day surgery remains underexplored.

Therefore, this study aimed to develop and validate an ML-based prediction model for 30-day unplanned readmission following lobectomy or sublobectomy in a day surgery pathway, and to identify key risk factors to guide targeted postoperative management.

## 2 Materials and methods

### 2.1 Data source

Data for this study were collected from patients who underwent surgery in Nursing Department of Affiliated Second Hospital, School of Medicine, Zhejiang University in Hangzhou, China, from December 2022 to January 2025. This study was approved by the Affiliated Second Hospital, School of Medicine, Zhejiang University in Hangzhou, China Ethics Committee [approval number: (2023) Research Ethics Approval No. (1070)]. The requirement for written informed consent from patients was waived in this study due to its retrospective design.

### 2.2 Inclusion and exclusion criteria

Inclusion criteria: (1) compliance with the Day Surgery Management Specifications formulated by the Medical Affairs Department of our hospital, including aged 18–75 years, clear consciousness, and no history of mental illness; (2) pathological diagnosis of lung cancer; (3) patients who underwent lobectomy or sublobectomy in the Department of Thoracic Surgery of our hospital, including lobectomy, anatomical segmentectomy, and non-anatomical sublobectomy/wedge resection; (4) complete baseline data, perioperative clinical data, imaging or laboratory test results, etc.; (5) available postoperative follow-up information and data on whether unplanned readmission occurred within 30 days.

Exclusion criteria: (1) patients not managed according to the day surgery model preoperatively or converted to the conventional hospitalization pathway for various reasons; (2) patients requiring long-term Intensive Care Unit (ICU) treatment due to severe intraoperative/postoperative complications, hospital stay significantly exceeding the ambulatory pathway (hospital stay > 2 days), or who died during the initial hospitalization; (3) patients directly transferred to other medical institutions for long-term hospitalization upon discharge; (4) no effective follow-up method or missing key outcome data after discharge.

### 2.3 Definition of 30-day unplanned readmission

Thirty-day unplanned readmission refers to an unforeseeable readmission within 30 days after the completion of the previous hospitalization, where the reason for readmission is the same or a related disease (13).

### 2.4 Data extraction and processing

The study variables included sex (female, male), age, payment category (medical insurance, rural insurance, self-paid), education level (college and above, high school, illiterate, junior high school, primary school, technical secondary school), marital status (divorced, married, single, widowed), smoking status (no, yes), alcohol consumption (no, yes), body mass index (BMI), prothrombin time (PT), D-dimer, glucose, white blood cell count (WBC), C-reactive protein (CRP), hemoglobin, platelet count (PLT), hospitalization duration, intraoperative blood loss, intraoperative blood transfusion volume, surgical approach (lobe, segment), staging (I, II, III), pathological diagnosis (adenocarcinoma, squamous cell carcinoma, other lung cancer), tumor count, surgical duration, tumor size, occupational category (farmers, staff/management/professionals, workers/manual laborers, unemployed/homemakers, business/service/self-employed, retired, unknown, other), forced vital capacity (FVC), FEV_1_, FEV_1_/FVC%, and diffusing capacity of the lung for carbon monoxide (single breath) (DLCO SB).

WBC, CRP, hemoglobin, and PLT were all measured on the first postoperative day.

### 2.5 Propensity score matching

To control for potential confounding effects arising from the choice of surgical approach (lobectomy vs. sublobectomy) —a core factor influencing perioperative pathways and outcomes—we performed propensity score matching (PSM) prior to modeling. Using a 1:3 matching ratio, we constructed a balanced cohort for analysis. It is important to note that PSM was employed here for cohort construction and to reduce heterogeneity related to surgical approach, not for causal inference. Consequently, the post-matching cohort represents a risk-enriched sample and does not reflect the real-world incidence of readmission.

### 2.6 ML feature selection, modeling, evaluation, and interpretability

The least absolute shrinkage and selection operator (LASSO) algorithm was used to assess the importance of candidate variables to further reduce multicollinearity and optimize the variable construction of ML models.

Data were split into a training set (70%) and a validation set (30%), with five-fold cross-validation performed to enhance model robustness.

Nine ML algorithms were utilized for model development: Logistic Regression (LR), eXtreme Gradient Boosting (XGBoost), light gradient boosting machine (LightGBM), ridge regression (RR), decision Tree (DT), K-nearest neighbors (KNN), random forest (RF), multi-layer perceptron (MLP), and support vector machine (SVM).

Model performance was evaluated using the area under the receiver operating characteristic curve (ROC-AUC), accuracy, kappa coefficient, sensitivity, specificity, F1-score, Precision-Recall Area Under Curve (PR-AUC), decision curve analysis (DCA), and calibration curves.

**FIGURE 1:**
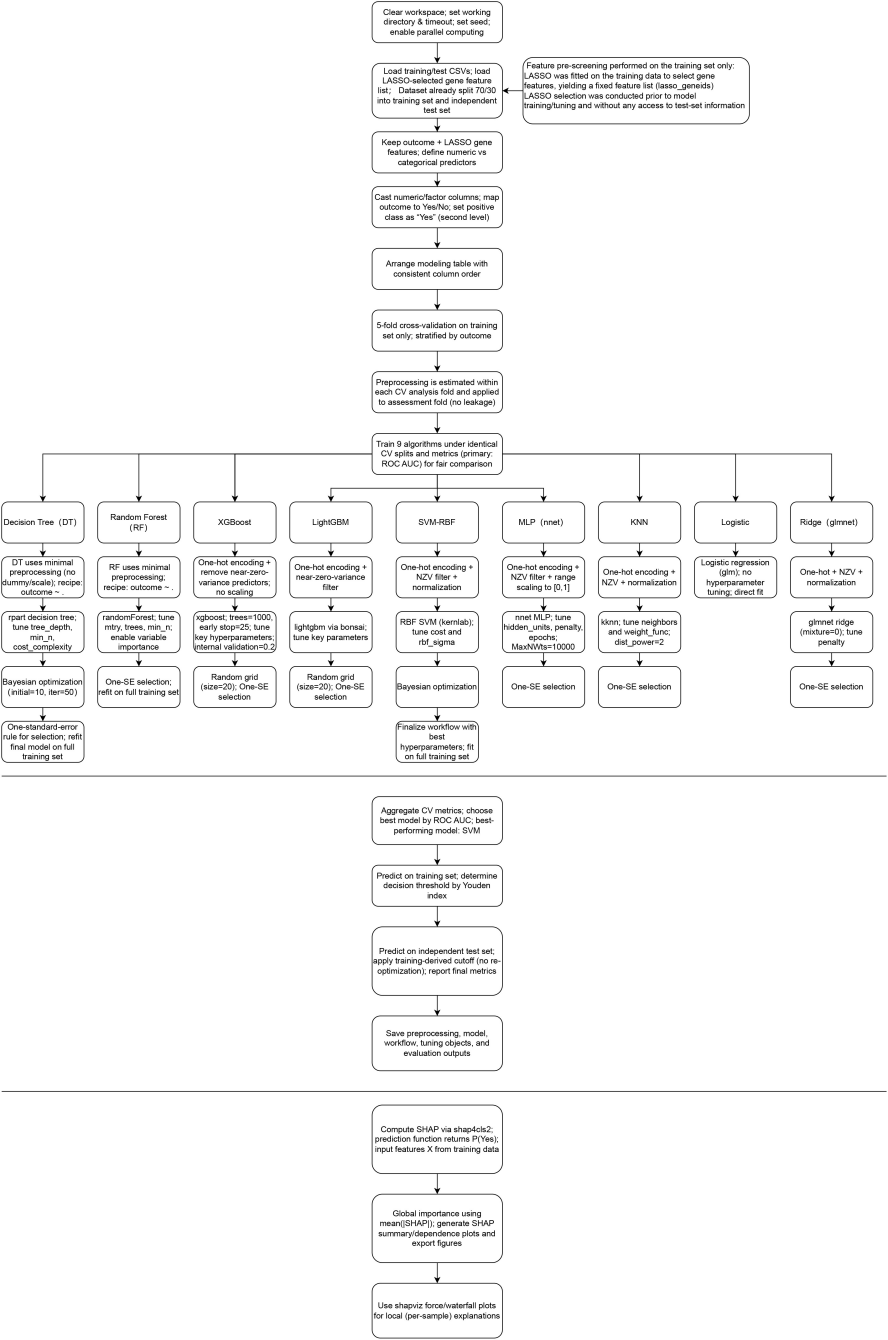
Flowchart of this study.

The interpretability of the models was visualized using SHapley Additive exPlanations (SHAP) plots. The flowchart for this study was shown in Figure 1.

### 2.7 Statistical analysis

R version 4.3.3 was used for data cleaning, analysis, and visualization. Normally distributed quantitative data were expressed as mean ± standard deviation, and intergroup comparisons were performed using analysis of variance. Non-normally distributed quantitative data were presented as median (interquartile range, IQR), and intergroup comparisons were conducted using the Kruskal-Wallis test. Qualitative data were expressed as counts (percentages), and intergroup comparisons were made using the chi-square test or Fisher’s exact test. A two-tailed *P-*value < 0.05 was considered statistically significant.

## 3 Results

### 3.1 Baseline characteristics of patients

Before PSM, a total of 8,320 patients were included in this study, of whom 127 experienced 30-day unplanned readmission; after PSM, a total of 380 patients were included in this study, of whom 111 experienced 30-day unplanned readmission (Table 1). The 30-day unplanned readmission group had a mean age of 60.89 ± 10.23 years, with a predominance of female patients and self-paid payment category. The control group had a mean age of 54.79 ± 13.17 years, with 68.0% female patients and 71.7% covered by medical insurance. Significant differences were observed between the two groups in age, payment category, marital status, smoke, BMI, PT, D-dimer, WBC, hemoglobin, PLT, intraoperative blood loss, surgical approach, pathological diagnosis, tumor count, tumor size, occupational category, FVC, and FEV_1_.

**TABLE 1:** Baseline characteristics of patients.

Variables	Level	Control group	30-Day unplanned readmission group	*P*
N		269	111	
Sex (%)	Female	183 (68.0)	70 (63.1)	0.416
	Male	86 (32.0)	41 (36.9)	
Age [mean (SD)]		54.79 (13.17)	60.89 (10.23)	< 0.001
Payment category (%)	Medical insurance	193 (71.7)	49 (44.1)	< 0.001
	Rural insurance	17 (6.3)	10 (9.0)	
	Self-paid	59 (21.9)	52 (46.8)	
Education (%)	College and above	73 (27.1)	18 (16.2)	0.072
	High school	40 (14.9)	16 (14.4)	
	Illiterate	20 (7.4)	18 (16.2)	
	Junior high school	55 (20.4)	24 (21.6)	
	Primary school	71 (26.4)	31 (27.9)	
	Technical secondary school	10 (3.7)	4 (3.6)	
Marital status (%)	Divorced	6 (2.2)	0 (0.0)	0.023
	Married	241 (89.6)	109 (98.2)	
	Single	15 (5.6)	0 (0.0)	
	Widowed	7 (2.6)	2 (1.8)	
Smoke (%)	No	219 (81.4)	75 (67.6)	0.005
	Yes	50 (18.6)	36 (32.4)	
Alcohol (%)	No	209 (77.7)	80 (72.1)	0.300
	Yes	60 (22.3)	31 (27.9)	
BMI [mean (SD)]		23.52 (2.83)	24.80 (7.08)	0.013
PT [mean (SD)]		12.79 (0.60)	13.96 (7.70)	0.014
D dimer [mean (SD)]		455.84 (1377.42)	732.25 (584.30)	0.042
Glucose [mean (SD)]		5.26 (1.83)	5.79 (11.51)	0.459
WBC [mean (SD)]		7.31 (9.06)	11.07 (12.45)	0.001
CRP [mean (SD)]		10.42 (25.38)	10.51 (23.52)	0.973
Hemoglobin [mean (SD)]		134.80 (19.86)	128.85 (13.68)	0.004
PLT [mean (SD)]		210.28 (57.28)	196.07 (56.76)	0.028
Hospitalization duration [mean (SD)]		1.85 (0.36)	1.91 (0.29)	0.125
Intraoperative blood loss [mean (SD)]		14.11 (18.43)	26.08 (39.79)	< 0.001
Intraoperative blood transfusion volume [mean (SD)]		0.00 (0.00)	0.18 (1.90)	0.12
Surgical approach (%)	Lobe	131 (48.7)	77 (69.4)	< 0.001
	Segment	138 (51.3)	34 (30.6)	
Staging (%)	I	255 (94.8)	100 (90.1)	0.149
	II	13 (4.8)	11 (9.9)	
	III	1 (0.4)	0 (0.0)	
Pathological diagnosis (%)	Adenocarcinoma	258 (95.9)	97 (87.4)	0.001
	Squamous cell carcinoma	11 (4.1)	10 (9.0)	
	Other lung cancer	0 (0.0)	4 (3.6)	
Tumor count [mean (SD)]		1.19 (0.52)	1.35 (0.66)	0.010
Surgery duration [mean (SD)]		95.78 (95.64)	69.41 (273.68)	0.165
Tumor size [mean (SD)]		1.21 (0.73)	1.57 (0.74)	< 0.001
Occupational category (%)	Farmer	31 (11.5)	34 (30.6)	< 0.001
	Staff/management/professional	33 (12.3)	7 (6.3)	
	Worker/manual labor	9 (3.3)	10 (9.0)	
	Unemployed/homemaker	12 (4.5)	6 (5.4)	
	Business/service/self-employed	9 (3.3)	5 (4.5)	
	Retired	44 (16.4)	43 (38.7)	
	Unknown	117 (43.5)	0 (0.0)	
	Other	14 (5.2)	6 (5.4)	
FVC [mean (SD)]		105.77 (14.85)	100.52 (14.05)	0.002
FEV_1_ [mean (SD)]		101.95 (15.51)	94.88 (15.59)	< 0.001
FEV_1_/FVC% [mean (SD)]		96.21 (12.06)	96.83 (12.22)	0.650
DLCO SB [mean (SD)]		86.91 (14.22)	88.45 (17.84)	0.377

BMI, body mass index; PT, prothrombin time; WBC, white blood cell; CRP, C-reactive protein; PLT, platelet count; FVC, forced vital capacity; FEV_1_, forced expiratory volume in 1 second; DLCO SB, diffusing capacity of the lung for carbon monoxide (single breath).

### 3.2 Feature selection

**FIGURE 2:**
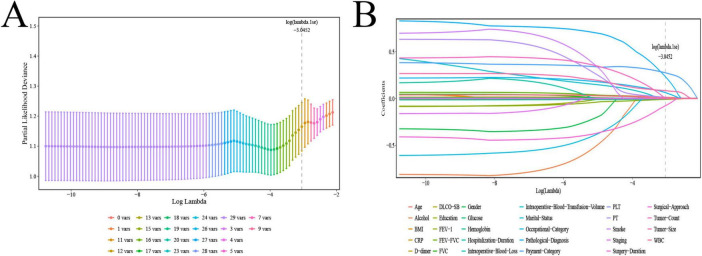
Feature variable selection by LASSO. LASSO, least absolute shrinkage and selection operator. **(A)** Number of non-zero coefficients in the model. **(B)** Number of variables corresponding to different λ values.

As shown in Figure 2A, the model bias reached the minimum and stabilized when log(λ) = –3.0452. Ultimately, 12 feature variables were selected for subsequent ML modeling, including age, payment category, PT, WBC, hemoglobin, intraoperative blood loss, surgical approach, pathological diagnosis, tumor count, tumor size, occupational category, and FEV_1_ (Figure 2B).

### 3.3 Model development and validation

**FIGURE 3:**
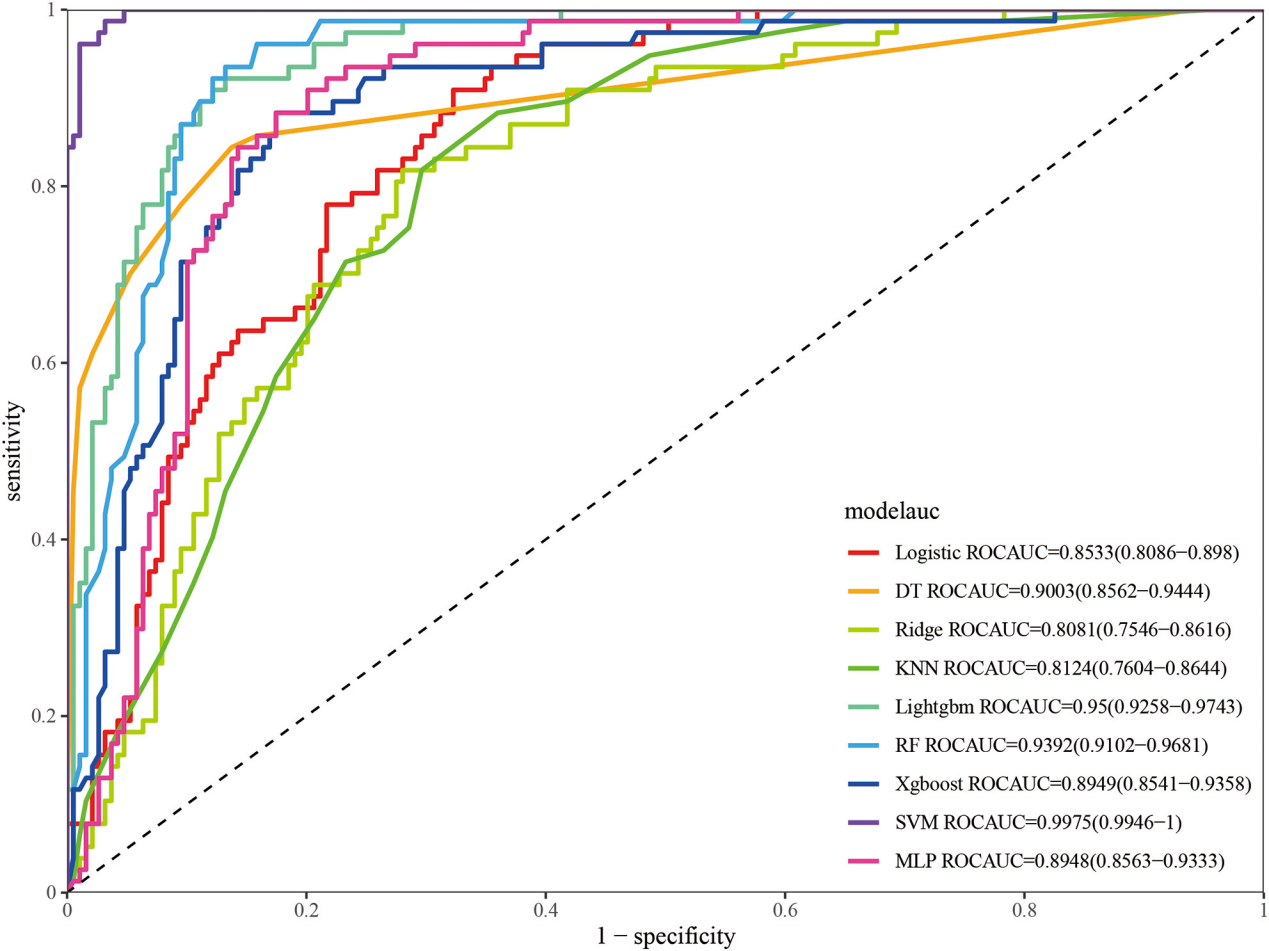
ROC curve of the training set. ROC, receiver operating characteristic; XGBoost, eXtreme gradient boosting; LightGBM, light gradient boosting machine; DT, decision tree; KNN, K-Nearest Neighbors; RF, random forest; MLP, multi-layer perceptron; SVM, support vector machine.

**FIGURE 4:**
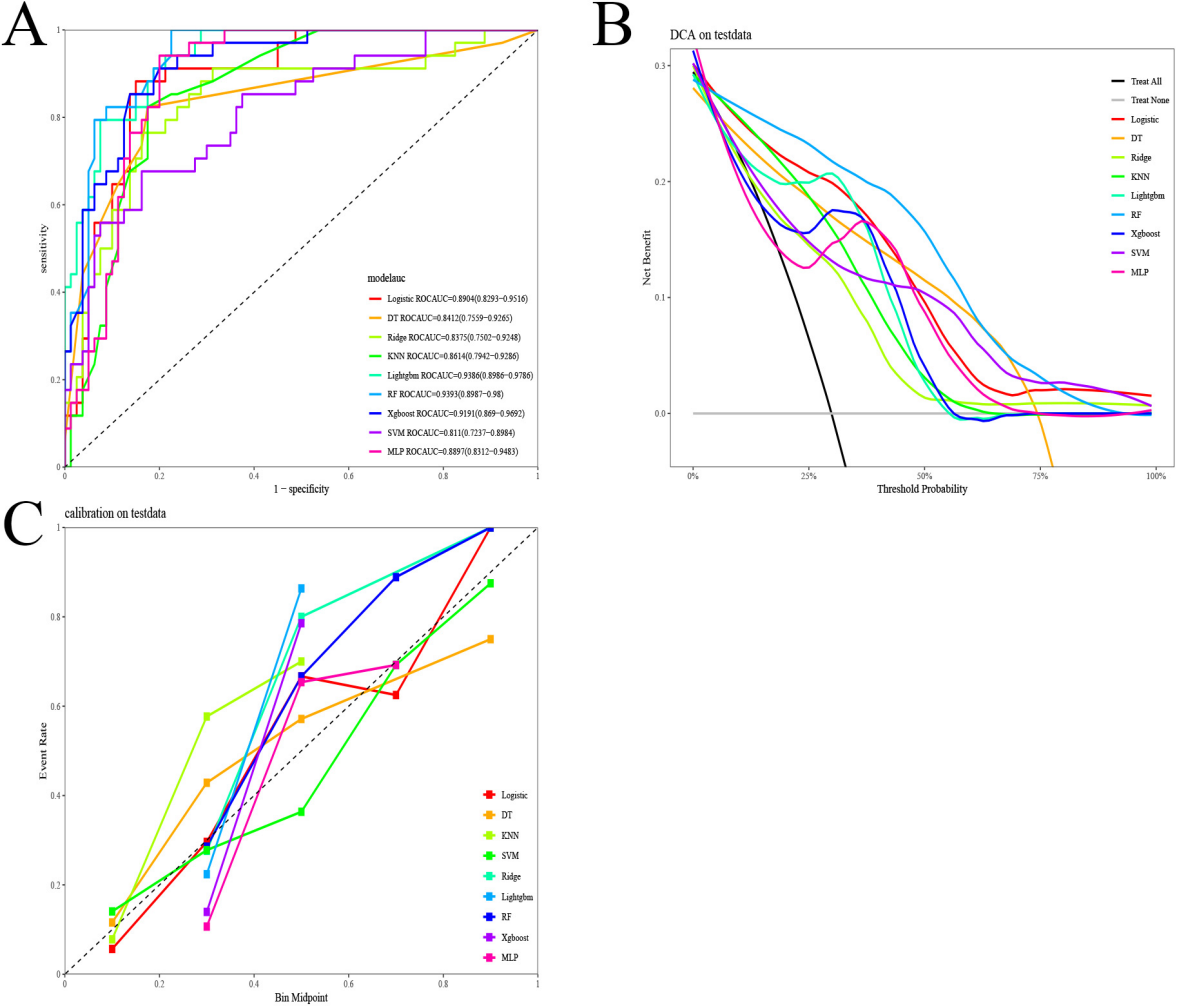
Model validation. **(A)** ROC curve of the validation set. **(B)** DCA (Decision Curve Analysis) curve of the validation set. **(C)** Calibration curve of the validation set. ROC, receiver operating characteristic; DCA, decision curve analysis; XGBoost, eXtreme gradient boosting; LightGBM, light gradient boosting machine; DT, decision tree; KNN, K-nearest neighbors; RF, random forest; MLP, multi-layer perceptron; SVM, support vector machine.

Nine ML algorithms were used to develop prediction models. As shown in Figure 3 and Table 2, the SVM model achieved the best performance in the training set, with an ROC-AUC of 0.997, PR-AUC of 0.994, accuracy of 0.974, kappa of 0.937, sensitivity of 0.987, specificity of 0.968, and F1-score of 0.956. In the validation set, RF and LightGBM performed relatively well (Figure 4 and Table 3). Specifically, the RF model achieved an ROC-AUC of 0.939, PR-AUC of 0.850, accuracy of 0.825, kappa of 0.631, sensitivity of 0.941, specificity of 0.775, and F1-score of 0.762; the LightGBM model achieved an ROC-AUC of 0.939, PR-AUC of 0.871, accuracy of 0.842, kappa of 0.652, sensitivity of 0.882, specificity of 0.825, and F1-score of 0.769. DCA results showed that within the threshold probability range of 10–80%, the RF model demonstrated favorable clinical net benefits, which were not only higher than those of the “treat all” and “treat none” strategies but also outperformed other ML models (Figure 4B). The calibration curve (Figure 4C) indicated that the predicted probabilities of the RF model were well-fitted to the actual event rates, being closer to the ideal reference line than other models, suggesting better predictive reliability. Based on ROC-AUC, accuracy, kappa, sensitivity, specificity, F1-score, PR-AUC, DCA, and calibration curves, the RF model was ultimately identified as the optimal prediction model.

**TABLE 2:** Predictive performance of nine machine learning models for 30-day unplanned readmission in the training set.

Model	Accuracy	Kappa	Sensitivity	Specificity	F1-score	ROC-AUC	PR-AUC
Logistic	0.744	0.485	0.909	0.677	0.673	0.853	0.648
DT	0.857	0.67	0.844	0.862	0.774	0.900	0.87
Ridge	0.748	0.468	0.818	0.720	0.653	0.808	0.562
KNN	0.711	0.426	0.883	0.640	0.638	0.812	0.573
LightGBM	0.883	0.736	0.922	0.868	0.821	0.95	0.871
RF	0.887	0.745	0.935	0.868	0.828	0.939	0.812
XGBoost	0.842	0.649	0.883	0.825	0.764	0.895	0.728
SVM	0.974	0.937	0.987	0.968	0.956	0.997	0.994
MLP	0.842	0.649	0.883	0.825	0.764	0.895	0.661

XGBoost, eXtreme gradient boosting; LightGBM, light gradient boosting machine; DT, decision tree; KNN, K-nearest neighbors; RF, random forest; MLP, multi-layer perceptron; SVM, support vector machine.

**TABLE 3:** Predictive performance of nine machine learning models for 30-day unplanned readmission in the validation set.

Model	Accuracy	Kappa	Sensitivity	Specificity	F1-score	ROC-AUC	PR-AUC
Logistic	0.825	0.619	0.882	0.800	0.75	0.89	0.732
DT	0.807	0.554	0.735	0.838	0.694	0.841	0.765
Ridge	0.807	0.554	0.735	0.838	0.694	0.838	0.704
KNN	0.789	0.550	0.853	0.762	0.707	0.861	0.600
LightGBM	0.842	0.652	0.882	0.825	0.769	0.939	0.871
RF	0.825	0.631	0.941	0.775	0.762	0.939	0.850
XGBoost	0.816	0.615	0.941	0.762	0.753	0.919	0.826
SVM	0.772	0.473	0.676	0.812	0.639	0.811	0.688
MLP	0.825	0.581	0.706	0.875	0.706	0.890	0.697

XGBoost, eXtreme gradient boosting; LightGBM, light gradient boosting machine; DT, decision tree; KNN, K-nearest neighbors; RF, random forest; MLP, multi-layer perceptron; SVM, support vector machine.

### 3.4 Model interpretability

**FIGURE 5:**
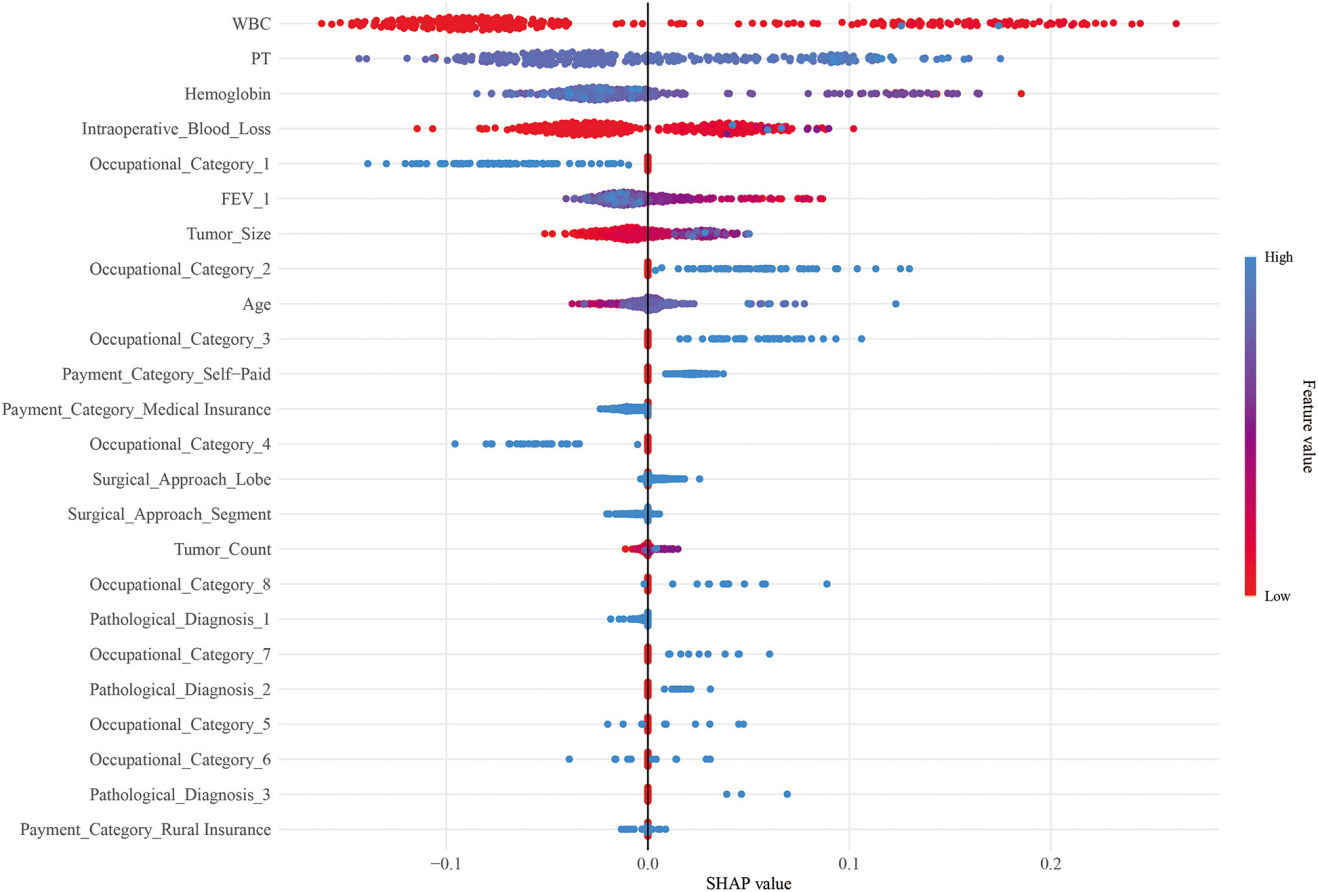
SHAP plots. SHAP, SHapley Additive exPlanations.

SHAP plots revealed that the top five factors in terms of feature importance were WBC, PT, hemoglobin, intraoperative blood loss, and unknown occupational categories (Figure 5). Among these, WBC, PT, and intraoperative blood loss were positively associated with 30-day unplanned readmission, hemoglobin were negatively associated with 30-day unplanned readmission.

## 4 Discussion

This study is the first to successfully construct and validate a ML model for predicting 30-day unplanned readmission risk in lung cancer patients undergoing lobectomy or sublobectomy under the day surgery model. The results indicate that the RF model exhibited the best predictive performance and clinical net benefits in the validation set, with key predictive factors including WBC, PT, hemoglobin, intraoperative blood loss, and unknown occupational category. These findings provide important evidence for early identification and precise intervention of high-risk patients in the context of the growing popularity of enhanced recovery after surgery and day surgery (14).

Unplanned readmission is a key indicator of medical quality and patient safety, closely associated with higher medical costs and poorer long-term prognosis (15). In previous studies on lung cancer surgery, various risk factors for unplanned readmission have been identified, such as decreased lung function and open surgery (8). However, these studies were mostly based on the traditional hospitalization model (hospital stay usually exceeding 5–7 days), and there is significant doubt whether their conclusions can be directly generalized to day surgery patients with extremely short hospital stays (≤ 2 days). The day surgery model aims to reduce medical resource utilization and accelerate recovery by optimizing perioperative processes (16), but it also means that patients may still be in the early postoperative recovery stage at discharge, with some potential complications not yet fully manifested. Therefore, research on readmission risk for this specific population is particularly necessary.

This study compared nine ML algorithms and ultimately determined RF as the optimal model. In the validation set, the RF model achieved excellent performance with an ROC-AUC of 0.939 and sensitivity of 0.941, and its DCA showed significant clinical net benefits within the threshold probability range of 10–80%. Consistent with our findings, RF has been shown to perform well in prognostic prediction in multiple studies. A study on patients with peripheral artery disease reported that among RF, SVM, MLP, XGBoost, RF achieved the best performance in predicting 30-day unplanned readmission after endovascular intervention for PAD, with an AUC of 0.66 (17). Similar results were found in a study on 30-day unplanned readmission after total shoulder arthroplasty (18). The excellent performance of the RF model in this study is mainly attributed to the compatibility between its algorithmic characteristics and the data features of this study. Firstly, as an ensemble learning algorithm, RF effectively reduces the overfitting risk of single decision trees by constructing a large number of decision trees and aggregating their results, thereby achieving more stable performance and stronger generalization ability in the validation set (19). Secondly, the predictive variables included in this study may have complex non-linear relationships and interactions. RF can naturally handle mixed-type data and automatically capture these non-linear relationships and interaction effects without the need for preset parameters or transformations like logistic regression (20). Finally, RF performs internal validation through out-of-bag error estimation (21) and provides feature importance ranking, offering good model interpretability while pursuing high prediction accuracy, which is crucial for clinical translation. To mitigate the risk of overfitting and enhance the generalization ability of the model, a multi-faceted strategy was adopted in this study. First, during the model training phase, strict five-fold cross-validation was applied to optimize model parameters (22) and evaluate its stability. This approach enables more efficient utilization of limited data and prevents the model from over-reliance on the specific sample distribution of the training set, serving as a well-recognized technique for overfitting prevention (23). Second, the SHAP framework was adopted to interpret model predictions. SHAP values not only generate a clear ranking of feature importance but also enable the visualization of relationships between features and predictive outcomes (24). Through the analysis of SHAP summary plots, we could verify whether model decisions were based on sound clinical logic (25) rather than data noise, which helps identify potential signs of overfitting.

SHAP plots revealed that WBC, PT, hemoglobin, intraoperative blood loss, and unknown occupational category were important features for 30-day unplanned readmission in this population, among which WBC, PT, and intraoperative blood loss were positively associated with 30-day unplanned readmission risk and hemoglobin were negatively associated with 30-day unplanned readmission. Elevated WBC often indicates potential infection or inflammatory responses (26, 27), while hemoglobin levels directly reflect the patient’s oxygen-carrying capacity (28). Both are sensitive indicators of the patient’s systemic condition. Under the day surgery model, abnormalities in WBC and hemoglobin on the first postoperative day can promptly detect signals of early postoperative physiological disturbance. Prolonged PT may reflect subclinical liver dysfunction (29) or consumption of coagulation factors (30), which could be associated with surgical stress, underlying nutritional status, and other contributing factors. These conditions may not only increase the risk of postoperative bleeding but also correlate with systemic inflammatory responses (31), thereby collectively elevating the risk of 30-day unplanned readmission. For day surgery patients lacking close monitoring after discharge, even mild delayed bleeding may lead to serious consequences and trigger readmission (32). Intraoperative blood loss directly reflects the severity and complexity of surgical trauma. Patients with significant blood loss are more prone to hypovolemia, reduced tissue perfusion, and may experience more severe systemic inflammatory responses, thereby increasing the risk of cardiopulmonary complications and infections (33). Different occupations may imply differences in education level, economic status, health literacy, working environment, and accessibility to subsequent medical resources (34, 35). We speculated that patients in the unknown occupational category in this study may have relatively insufficient home care resources, poor postoperative wound care and medication adherence, and limited ability to identify abnormal postoperative symptoms, further increasing the risk of 30-day unplanned readmission. Notably, some traditional risk factors identified in previous studies, such as age and lung function indicators, were retained in LASSO selection but ranked relatively low in SHAP analysis. This may be because the strict selection criteria for day surgery have excluded patients with extremely poor lung function or advanced age and frailty, making indicators reflecting acute physiological disorders (WBC, PT) and immediate surgical trauma stronger risk signals in this relatively homogeneous population.

The model constructed and findings obtained in this study provide direct guidance for the clinical nursing practice of ambulatory lung cancer surgery and also facilitate the transformation of nursing models from an experience-driven approach to data-driven precision nursing. First, this study identified and optimized the core focus of perioperative nursing monitoring, indicating that nursing practice should emphasize the accurate documentation and assessment of intraoperative blood loss, as well as the dynamic analysis of early postoperative routine blood test and coagulation function results. Even if these indicators fall within the clinically acceptable normal range, their changing trends or values approaching the upper limit of the normal range should be regarded as early warning signals for nursing staff. Second, the model enables preoperative risk stratification and the formulation of personalized discharge plans: the nursing team can conduct rapid risk assessments using the model integrated with key predictive factors, and for screened high-risk patients, develop refined discharge guidance focusing on the monitoring of WBC, PT, hemoglobin and blood loss. Meanwhile, transitional nursing resources are coordinated to achieve doctor-patient joint tracking through the internet-based nursing service platform, and nursing staff take the initiative to connect with community nursing services and family doctors to ensure the continuity of nursing care. In addition, the study findings provide practical support for nurse-led post-discharge follow-up. In the future, the model can be further improved by expanding the sample size and conducting multicenter validation studies. It is expected to be integrated into the electronic health record system to develop a digital decision support tool for nursing staff, which can automatically generate risk scores and personalized nursing checklists upon patient discharge, thereby improving the timeliness and effectiveness of nursing interventions.

Although this study provides new evidence for the unplanned readmission management of lung cancer patients undergoing day surgery, it has certain limitations. Firstly, the data were derived from a single hospital in Zhejiang Province, and the homogeneity of patients’ regional and socioeconomic characteristics may lead to selection bias. The external validity of the model needs to be verified by multi-center, large-sample studies. Secondly, this study did not include social-psychological factors such as the patient’s home care support level and postoperative medication adherence, which may affect postoperative recovery and unplanned readmission risk in ambulatory patients. Third, this study only focused on unplanned readmission within 30 days, while long-term complications in day surgery patients may lead to readmission beyond this period. Finally, although cross-validation was implemented in the training set and model performance was evaluated in an independent validation set in this study, the limited number of outcome events may compromise the stability and reproducibility of some machine learning models, with a certain residual risk of overfitting remaining. Future studies should conduct external validation and necessary model recalibration in larger-sample, multi-center cohorts to evaluate their generalization ability and verify the stability of feature importance (including SHAP interpretation); meanwhile, extend the follow-up period to improve the prediction dimension of the model, supplement social-psychological variables, and construct dynamic prediction models to achieve continuous monitoring and intervention of perioperative risks.

Future studies should extend the follow-up period to improve the prediction dimension of the model. Additionally, future research can further include multi-center data of lung cancer patients undergoing day surgery, supplement social-psychological variables, and construct dynamic prediction models to achieve continuous monitoring and intervention of perioperative risks.

## 5 Conclusion

In summary, this study successfully constructed a ML prediction model for 30-day unplanned readmission in lung cancer patients undergoing day surgery. The RF model performed excellently, and its identified key risk factors: WBC, PT, hemoglobin, intraoperative blood loss, and unknown occupational category, provide clear intervention targets for clinical practice, especially nursing. This study is of great practical significance for implementing targeted, intensity-stratified transitional care, safeguarding patient safety, improving medical quality, and optimizing resource allocation.

## Data Availability

The raw data supporting the conclusions of this article will be made available by the authors, without undue reservation.

## References

[1] Bray F Laversanne M Sung H Ferlay J Siegel RL Soerjomataram I Global cancer statistics 2022: globocan estimates of incidence and mortality worldwide for 36 cancers in 185 countries. *CA Cancer J Clin*. (2024) 74:229–63. 10.3322/caac.21834 38572751

[2] Dziedzic R Marjański T Rzyman W. A narrative review of invasive diagnostics and treatment of early lung cancer. *Transl Lung Cancer Res*. (2020) 10:1110–23. 10.21037/tlcr-20-728 33718049 PMC7947400

[3] Petrella F Cara A Cassina EM Degiovanni S Libretti L Pirondini E The role of sublobar resection in early-stage non-small-cell lung cancer. *J Clin Med*. (2024) 13:5277. 10.3390/jcm13175277 39274490 PMC11396031

[4] Wu S Wu H Chen T Shen J. ASO author reflections: survival outcomes of sublobectomy and lobectomy for elderly patients with peripheral solid-dominant non-small cell lung cancer. *Ann Surg Oncol*. (2023) 30:1530–1. 10.1245/s10434-022-13015-9 36581720

[5] Li Z Xia M Liu C Wang T Ren Y Liu Y. A meta-analysis of minimally invasive surgery versus thoracotomy for centrally located non-small cell lung cancer. *J Thorac Dis*. (2021) 13:252–61. 10.21037/jtd-20-3273 33569205 PMC7867798

[6] Bottet B Piton N Selim J Sarsam M Guisier F Baste JM. Beyond the frontline: a triple-line approach of thoracic surgeons in lung cancer management-state of the art. *Cancers*. (2023) 15:4039. 10.3390/cancers15164039 37627067 PMC10452134

[7] Prisciandaro E Bertolaccini L Passani S Szanto Z. Predictors of 30-day morbidity and unplanned readmission after induction therapy followed by lung resection for non-small cell lung cancer: European society of thoracic surgeons database analysis. *Eur J Cardiothorac Surg*. (2025) 67:ezaf426. 10.1093/ejcts/ezaf426 41308163

[8] Pons A Guirao Á Fibla JJ Carvajal C Embun R Sánchez D National evaluation of risk factors for unplanned readmission after lung resection. *Eur J Cardiothorac Surg*. (2022) 61:1251–7. 10.1093/ejcts/ezac081 35218337

[9] Bouabdallah I Pauly V Viprey M Orleans V Fond G Auquier P Unplanned readmission and survival after video-assisted thoracic surgery and open thoracotomy in patients with non-small-cell lung cancer: a 12-month nationwide cohort study. *Eur J Cardiothorac Surg*. (2021) 59:987–95. 10.1093/ejcts/ezaa421 33236091

[10] Kim JH Cheon BR Kim MG Hwang SM Lim SY Lee JJ Harnessing machine learning for prediction of postoperative pulmonary complications: retrospective cohort design. *J Clin Med*. (2023) 12:5681. 10.3390/jcm12175681 37685748 PMC10488713

[11] Bellini V Valente M Bertorelli G Pifferi B Craca M Mordonini M Machine learning in perioperative medicine: a systematic review. *J Anesth Analg Crit Care*. (2022) 2:2. 10.1186/s44158-022-00033-y 37386544 PMC8761048

[12] Sukhadia SS Muller KE Workman AA Nagaraj SH. Machine learning-based prediction of distant recurrence in invasive breast carcinoma using clinicopathological data: a cross-institutional study. *Cancers*. (2023) 15:3960. 10.3390/cancers15153960 37568776 PMC10416932

[13] Li J Hou YX Wm Q. Risk prediction models for unplanned readmission in cancer patients: a scoping review. *Chin J Nurs.* (2022) 57:1079–87. 10.3761/j.issn.0254-1769.2022.09.008

[14] Yang J Ge L Ju XX Liu XX. Status and influencing factors of discharge readiness in day surgery lung cancer patients under a fast-track rehabilitation pathway. *J Clin Nurs*. (2025) 34:4779–87. 10.1111/jocn.17743 40211487

[15] Ellul S Shoukry M. The impact of unplanned 30-day readmission as a quality indicator in pediatric surgery. *Front Surg*. (2023) 10:1199659. 10.3389/fsurg.2023.1199659 37325416 PMC10264661

[16] Tan KM Xu X Wj C. “Pulmonary department experience” in the exploration of ambulatory surgery for pulmonary tumors: Taking video-assisted thoracoscopic wedge resection of the lung and pulmonary bulla resection as examples. *Chin Health Qual Manage.* (2025) 32:10–23. 10.13912/j.cnki.chqm.2025.32.7.03

[17] Cox M Panagides JC Tabari A Kalva S Kalpathy-Cramer J Daye D. Risk stratification with explainable machine learning for 30-day procedure-related mortality and 30-day unplanned readmission in patients with peripheral arterial disease. *PLoS One*. (2022) 17:e0277507. 10.1371/journal.pone.0277507 36409699 PMC9678279

[18] Arvind V London DA Cirino C Keswani A Cagle PJ. Comparison of machine learning techniques to predict unplanned readmission following total shoulder arthroplasty. *J Shoulder Elbow Surg*. (2021) 30:e50–9. 10.1016/j.jse.2020.05.013 32868011

[19] Digumarthi V Amin T Kanu S Mathew J Edwards B Peterson LA Preoperative prediction model for risk of readmission after total joint replacement surgery: a random forest approach leveraging NLP and unfairness mitigation for improved patient care and cost-effectiveness. *J Orthop Surg Res*. (2024) 19:287. 10.1186/s13018-024-04774-0 38725085 PMC11084055

[20] Couronné R Probst P Boulesteix AL. Random forest versus logistic regression: a large-scale benchmark experiment. *BMC Bioinformatics*. (2018) 19:270. 10.1186/s12859-018-2264-5 30016950 PMC6050737

[21] Janitza S Hornung R. On the overestimation of random forest’s out-of-bag error. *PLoS One*. (2018) 13:e0201904. 10.1371/journal.pone.0201904 30080866 PMC6078316

[22] Wang D Li Y Wang L. Enhancing clinical decision-making in closed pelvic fractures with machine learning models. *Biomol Biomed*. (2025) 25:1491–507. 10.17305/bb.2024.10802 39652135 PMC12097389

[23] Li Q Wei X Wu F Qin C Dong J Chen C Development and validation of preeclampsia predictive models using key genes from bioinformatics and machine learning approaches. *Front Immunol*. (2024) 15:1416297. 10.3389/fimmu.2024.1416297 39544937 PMC11560445

[24] Lee J Park KM Park S. Interpretable machine learning for prediction of clinical outcomes in acute ischemic stroke. *Front Neurol*. (2023) 14:1234046. 10.3389/fneur.2023.1234046 37745661 PMC10513028

[25] Hiraga K Takeuchi M Kimura T Yoshida S Kawakami K. Prediction models for in-hospital deaths of patients with COVID-19 using electronic healthcare data. *Curr Med Res Opin*. (2023) 39:1463–71. 10.1080/03007995.2023.2270420 37828849

[26] Zhai X Feng M Guo H Liang Z Wang Y Qin Y Development of prediction models for new integrated models and a bioscore system to identify bacterial infections in systemic lupus erythematosus. *Front Cell Infect Microbiol*. (2021) 11:620372. 10.3389/fcimb.2021.620372 33732661 PMC7957015

[27] Imiela AM Mikołajczyk TP Pruszczyk P. Novel insight into inflammatory pathways in acute pulmonary embolism in humans. *Arch Immunol Ther Exp.* (2024) 72:21. 10.2478/aite-2024-0021 39466143

[28] Dubey Y Mange P Barapatre Y Sable B Palsodkar P Umate R. Unlocking precision medicine for prognosis of chronic kidney disease using machine learning. *Diagnostics*. (2023) 13:3151. 10.3390/diagnostics13193151 37835894 PMC10572800

[29] Lee SW Lee HC Suh J Lee KH Lee H Seo S Multi-center validation of machine learning model for preoperative prediction of postoperative mortality. *NPJ Digit Med*. (2022) 5:91. 10.1038/s41746-022-00625-6 35821515 PMC9276734

[30] Harris NS Marin MJ Butenas S. Beyond prothrombin time and activated partial thromboplastin time: coagulation in vivo-an illustrated review. *Lab Med*. (2025) 56:438–47. 10.1093/labmed/lmae125 40319459

[31] Liu Y Gao W Guo W Guo Y Shi M Dong G Prominent coagulation disorder is closely related to inflammatory response and could be as a prognostic indicator for ICU patients with COVID-19. *J Thromb Thrombolysis*. (2020) 50:825–32. 10.1007/s11239-020-02174-9 32761495 PMC7408978

[32] Merkow RP Ju MH Chung JW Hall BL Cohen ME Williams MV Underlying reasons associated with hospital readmission following surgery in the United States. *JAMA*. (2015) 313:483–95. 10.1001/jama.2014.18614 25647204

[33] Li S Zhou K Lai Y Shen C Wu Y Che G. Estimated intraoperative blood loss correlates with postoperative cardiopulmonary complications and length of stay in patients undergoing video-assisted thoracoscopic lung cancer lobectomy: a retrospective cohort study. *BMC Surg*. (2018) 18:29. 10.1186/s12893-018-0360-0 29792183 PMC5966911

[34] Alqarni AS Pasay-An E Saguban R Cabansag D Gonzales F Alkubati S Relationship between the health literacy and self-medication behavior of primary health care clientele in the Hail Region, Saudi Arabia: implications for Public Health. *Eur J Investig Health Psychol Educ*. (2023) 13:1043–57. 10.3390/ejihpe13060080 37366784 PMC10297701

[35] Akimova ET Wolfram T Ding X Tropf FC Mills MC. Polygenic prediction of occupational status GWAS elucidates genetic and environmental interplay in intergenerational transmission, careers and health in UK Biobank. *Nat Hum Behav*. (2025) 9:391–405. 10.1038/s41562-024-02076-3 39715877 PMC11860221

